# Chemical Analysis of the Essential Oil from *Siparuna echinata* (Kunth) A. DC. (Siparunaceae) of Ecuador and Isolation of the Rare Terpenoid Sipaucin A

**DOI:** 10.3390/plants9020187

**Published:** 2020-02-04

**Authors:** Jessica García, Gianluca Gilardoni, Nixon Cumbicus, Vladimir Morocho

**Affiliations:** 1 Departamento de Química y Ciencias Exactas, Universidad Técnica Particular de Loja (UTPL), Loja 1101608, Ecuador; jkgarcia3@utpl.edu.ec (J.G.); gianluca.gilardoni@gmail.com (G.G.); 2 Departamento de Ciencias Biológicas, Universidad Técnica Particular de Loja (UTPL), Loja 1101608, Ecuador; nlcumbicus@utpl.edu.ec

**Keywords:** *Siparuna echinata*, essential oil, enantioselective analysis, Sipaucin A, Ecuador

## Abstract

The phytochemical research on the species *Siparuna echinata*, collected in the Province of Loja (Ecuador), led to the isolation of a rare sesquiterpenoid, called Sipaucin A. The structure was confirmed by nuclear magnetic resonance (NMR) and mass spectrometry (MS). Furthermore, the essential oil of the fruits was obtained by hydrodistillation and analyzed by gas chromatography, coupled to mass spectrometry (GC-MS) and flame ionization detector (GC-FID). Twenty-seven compounds were identified in a polydimethylsiloxane column (DB-5ms) and nineteen in a polyethylene glycol column (HP-INNOWax). Major compounds were α-pinene (24.3%, 20.3%), β-pinene (21.7%, 22.7%), β-myrcene (11.3%, 14.8%), limonene (10.0%, 11.3%), *cis*-ocimene (8.5%, 8.1%), and *trans*-ocimene (8.9%, 8.4%). In addition to the chemical analysis, the essential oil was submitted to enantioselective analysis of two major chiral monotherpenes, determining an enantiomeric excess of 100.0% for (+)-α-pinene and 6.7% for (+)-β-pinene.

## 1. Introduction

The geographic location of Ecuador, together with its geological features, makes this region one of the richest of the world in biodiversity. According to the WCMC (World Conservation Monitoring Center) of the United Nations, Ecuador is considered among the 17 megadiverse countries, counting with about 10% of the entire world plant species [1,2].

Every year, new plants are discovered and classified by botanists, while most of the known ones have never been studied so far for their metabolic composition. This fact converts Ecuador to an invaluable source of potentially new molecules of biological and pharmaceutical interest [3].

For all these reasons, the authors have been carrying on, since many years, the chemical description of the secondary metabolite profile of never studied species in Ecuador, especially in the Province of Loja. These studies are mainly focused on chemistry, with the structure elucidation of natural products, the analysis of new essential oils, and the description of biological activities [4,5,6,7].

In this work, we focused on some new terpenoids called Sipaucins, recently discovered in the species *Siparuna pauciflora* and described so far only once in literature [8]. Furthermore, *Siparuna* spp. often produce a volatile fraction that appeared to be abundant in fruits of *S. echinata*, justifying the study of the essential oil (EO).

The Siparunaceae, previously considered as part of the Monimiaceae, include two genera: *Glossocalix* and *Siparuna*. They have been reported for the presence of sesquiterpene volatile fractions, showing the property of inducing cell apoptosis [9,10,11].

The genus *Siparuna* includes 235 plant species distributed in tropical regions of Central and South America [12]. It is characterized by biosynthesizing interesting secondary metabolites such as sesquiterpenes [13], flavonoids [13,14], and isoquinoline alkaloids [15,16], found in plant extracts and in essential oils, with a broad spectrum of pharmacological properties, such as: antibacterial, antimalarial, and antileishmaniasis [17].

*Siparuna echinata* is a native shrub of the Andean region, widespread from Colombia to Bolivia. It is known with the synonyms of *Citrosma apicifera* Tul., *Citrosma buddleiifolia* Benth., *Citrosma echinata* Kunth, *Siparuna apicifera* (Tul.) A. DC., *Siparuna buddleiifolia* (Benth.) A. DC., *Siparuna holtonii* A. DC., *Siparuna killipii* Perkins, *Siparuna olivaceo-velutina* Sleumer, *Siparuna phaneroneura* Standl., and *Siparuna valenzuelae* Cuatrec [18,19].

In Ecuador, it grows in the provinces of Bolivar, Carchi, Chimborazo, Cotopaxi, Imbabura, Morona Santiago, Napo, Pichincha, and Sucumbíos, between 1800 and 3650 m above sea level [14]. In this study, to the best of the authors’ knowledge, this species has been described for the first time in the Province of Loja. Commonly known as “limoncillo”, it is used by infusion of fruits and leaves in traditional medicine to treat digestive disorders and rheumatisms [2,3,4,5,6,7,8,9,10,11,12,13,14,15,16,17,18,19].

## 2. Results

### 2.1. Characterization of Sipaucin A

**Figure 1:**
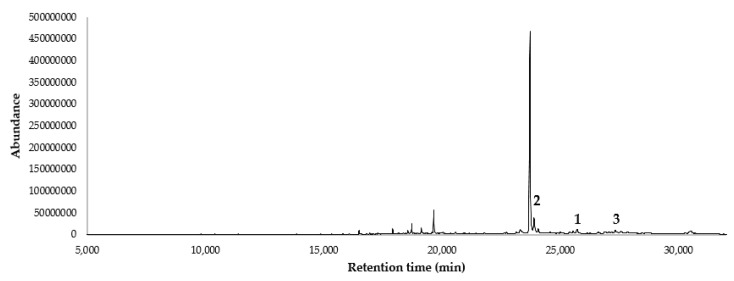
Selective gas chromatography–mass spectrometry (GC-MS) analysis (single ion monitoring, SIM) for Sipaucins ions in the ethyl acetate extract of *Siparuna echinata.* **1**, possible peak of Sipaucin A; **2**, possible peak of Sipaucin B; **3**, possible peak of Sipaucin C.

The ethyl acetate (EtOAc) extract of *S. echinata* led to the isolation and characterization of Sipaucin A. After removing chlorophyll, the extract was submitted to gas chromatography–mass spectrometry (GC-MS) analysis, based on a single ion monitoring (SIM) method, that was prepared according to the ions described in literature for the electron impact MS (EIMS) spectra of Sipaucins A–C [8]. The resulting chromatogram is shown in Figure 1.

**Figure 2:**
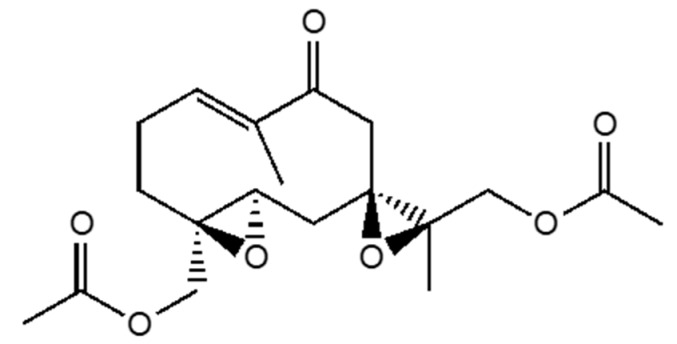
Structure of Sipaucin A.

Figure 1 evidenced the presence of some metabolites consistent with the ionization pattern of Sipaucins. The preparative fractionation of the extract achieved only Sipaucin A (Figure 2) with 0.3 % yield (*w*/*w* referred to chlorophyll-free mixture). It was submitted to NMR spectroscopy, resulting in data identical to those from literature [8].

### 2.2. Essential Oil Analysis

The EO distillation produced a yield of 1.09 ± 0.10 % (*w/w*, referred to dry fruits). In the chemical analysis of the EO of *S. echinata*, a total of twenty-seven and nineteen compounds were identified and quantified, respectively, with DB-5ms and HP-INNOWax columns. The EO was mainly composed of monoterpene hydrocarbons, corresponding to 84.8% and 89.7% of the total composition, respectively. Major compounds were α-pinene (24.3%, 20.3%), β-pinene (21.7%, 22.7%), β-myrcene (11.3%, 14.8%), limonene (10.0%, 11.3%), *cis*-ocimene (8.5%, 8.1%), and *trans*-ocimene (8.9%, 8.4%). The chemical composition of the EO is shown in Table 1 according to the elution order in DB-5ms.

#### Enantioselective GC Analysis

**Figure 3:**
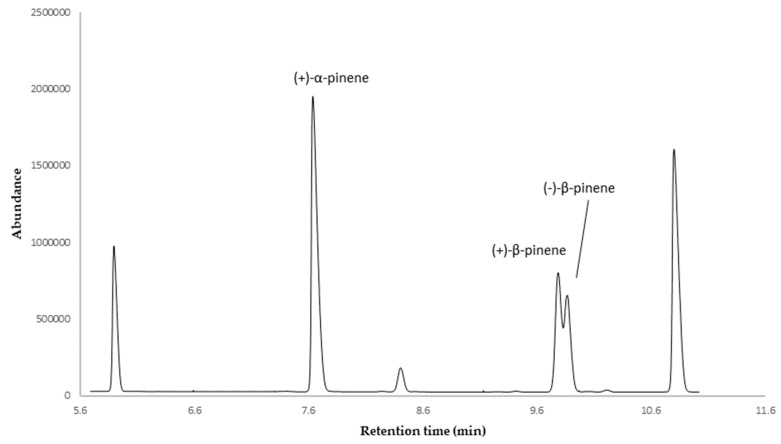
Enantioselective analysis of essential oil (EO) of *S. echinata* on diacethyl terbutylsilyl-β-cyclodextrin column.

The enantioselective analysis of the EO was carried out with a β-cyclodextrine-based GC column, coated with 2,3-diacethyl-6-tert-butylsilyl-β-cyclodextrin as chiral selector. The analysis was performed on two of the four major compounds (α-pinene and β-pinene), with myrcene being a non-chiral molecule and limonene enantiomers not being separable with this chiral selector. The results of the enantioselective analysis are shown in Table 2, expressed as enantiomeric distribution and enantiomeric excess (*ee*), and Figure 3.

## 3. Discussion

Sipaucins are rare sesquiterpenoids that have been described for the first time in 2003 in *S. pauciflora* [8] and not yet found in other species until now. As these metabolites could be characteristic of *Siparuna* genus, we decided to selectively search for them in the ethyl acetate extract, performing a single ion monitoring (SIM) GC-MS analysis. In the chromatogram of Figure 1, all the peaks consistent with the selected ions are shown. In particular, peak 1, 2, and 3 represent compounds whose ions and relative abundance are, respectively, compatible with Sipaucin A, B, and C. It should be underlined that these peaks are quite evident only in SIM analysis and splitless injection, demonstrating that they actually correspond to minor constituents of the extract. This fact could justify the low yield of Sipaucin A and the non-obtention of other Sipaucins. The presence of more than three peaks, all being compatible with this family of metabolites suggests that other new related compounds are possibly produced by *S. echinata*, though there is no evidence of the certain presence of these molecules in the extract.

Concerning the chemical composition of the volatile fraction, the EOs from other species of genus *Siparuna* were mainly based on sesquiterpenes: *S. aspera* and *S. macrotepala* presented as major compounds germacrene D (23.2%, 42.1%), bicyclogermacrene (7.8%, 11.8%), and α-pinene (7.0%, 1.8%) [34], while in the essential oil of *S. eggersii* the principal components were epicurzerenone (29.9%), germacrone (10.5%), and caryophyllene oxide (29.0%) [23]. On the other hand, our EO was mainly composed of monoterpenes, with α-pinene (24.3%), β-pinene (21.7%), β-myrcene (11.3), and limonene (10.0%) being major constituents.

Nevertheless, germacrenes are constantly present, which is an important factor, as they are structurally and biogenetically related [35] to Sipaucin A.

The enantioselective analysis showed (+)-α-pinene as a pure enantiomer, while (+)-β-pinene evidenced enantiomeric excess.

## 4. Materials and Methods

### 4.1. General Information

The NMR spectra were performed in a Varian spectrometer (Walnut Creek, CA, USA, 400 MHz for ^1^H and 100 MHz for ^13^C) in CDCl_3_ (from Sigma Aldrich, Saint Louis, MO, USA). The chemical shifts were reported in δ (ppm), relative to the signal of tetramethylsilane (TMS) and the coupling constants (*J*) in Hz.

The GC-MS analyses were performed on an Agilent Technologies (Wilmington, DE, USA) 6890N gas chromatograph coupled to a mass spectrometer detector (MSD) Agilent Technologies 5973N. For both GC-MS and gas chromatography–flame ionization detector (GC-FID) analyses, the instrument was equipped with a DB-5ms Agilent 122-5532 column (length 30 m, 0.25 mm internal diameter and 0.25 μm stationary phase thickness) and a HP-INNOWax column (length 30 m, 0.25 mm internal diameter and 0.25 μm stationary phase thickness). The MSD was equipped with an electron impact ion source, operating at 70 eV. The ion analyzer was operated in SCAN mode for the EO analysis (35–350 amu) and SIM mode for Sipaucins’ detection.

The enantioselective GC analysis was carried out with an enantioselective capillary column, based on 2,3-diacethyl-6-*tert*-butylsilyl-β-cyclodextrin as chiral selector (25 m × 0.25 mm × 0.25 μm, purchased from MEGA, Milan, Italy), installed in the same GC-MS system. Helium was used as carrier gas (Indura, Guayaquil, Ecuador).

Silica gel 60 (Merck KGaA, Darmstadt, Germany, from 0.063 to 0.200 mm) and RP-18 (Merck 40–63 μm) were used as stationary phases for column chromatography (CC). Normal phase thin layer chromatography (TLC) plates, with fluorescence indicator at 254 nm, were purchased from Sigma-Aldrich.

After exposure to UV light (254 and 366 nm), the plates were treated with a mixture of sulfuric acid and vanillin as chromatic reagent for spot detection.

All organic solvents for TLC and CC were technical grade, purchased from Relubquim Cia. Ltd.a. (Quito, Ecuador), and distilled before using. All solvents for GC, alkanes for linear retention indices (LRI), and the internal standard (*n*-nonane) were of analytical grade (purity > 99%), from Sigma-Aldrich. The calibration standard was isopropyl caproate, obtained in the authors’ laboratory by synthesis and purified to 98.8% (GC-FID purity).

### 4.2. Plant Material

The aerial parts of *S. echinata* were collected on May 2017 in the sector Guchanama, canton of Celica, province of Loja, with coordinates 4°05′31″ S and 79°57′18″ W, at 2220 m above sea level. The botanical sample was identified by one of the authors (N.C.) and deposited in the herbarium of the Universidad Técnica Particular de Loja, with voucher number VMZ-120. The samples were collected under governmental permission (MAE-DNB-CN-2016-0048).

### 4.3. Extraction and Isolation of Sipaucin A

The dried leaves of *S. echinata* (730 g) were extracted by Soxhlet with ethyl acetate (EtOAc) for 10 h. Soxhlet extraction was preferred to static maceration because, in a previous essay, the two methods were compared on analytical samples of plant material, producing the same chromatographic profile with higher yield in favor of Soxhlet. A yield of 27.4 % (*w/w*) was obtained.

An amount of 10 g of extract was eluted on C-18 reversed phase, in order to completely remove chlorophylls. The elution was performed isocratically with a mixture of methanol/water in the ratio of 85:15, producing 5 g of chlorophyll-free extract. To prove the presence of Sipaucins, 20 mg of chlorophyll-free extract was dissolved in 1 mL of dichloromethane and the solution (1 μL) was injected in splitless mode in GC-MS (DB-5ms column). The elution was conducted with a thermal gradient, according to the following program: the initial temperature was 50 °C maintained for 3 min, followed by a thermal gradient of 10 °C/min until the temperature of 250 °C, that was maintained for 17 min. The injector temperature was set at 250 °C and the carrier gas (helium) at 1 mL/min (constant flow). The SIM method is described in Table 3.

The preparative purification by CC was performed from 2 g of chlorophyll-free extract. Silica gel (200 g) was packed in a glass column (50 mm × 800 mm), and the extract was eluted in fractions with varying ratios of eluent mixture hexane/EtOAc from 100% hexane until 100% EtOAc. A total of 14 fractions were obtained (JK01-JK14), according to TLC profiles.

The fraction JK11 (134 mg) was further purified by CC in isocratic conditions, eluting with a mixture of hexane/EtOAc in the ratio of 95:5. A total of five fractions were collected according to TLC and subfraction 4 (15 mg) was subjected to preparative TLC. The plate was eluted with hexane/EtOAc in the ratio of 6:4, yielding 3 mg of pure Sipaucin A.

The metabolite was characterized by NMR spectroscopy, resulting in data identical to those from literature (see Supplementary Material, Figures S1 and S2 and Table S1).

### 4.4. Analysis of the EO

The volatile fraction was obtained by separately hydrodistilling four amounts of 265 g of dried fruits for 5 h, using a Clevenger type glass apparatus. The essential oil was collected and dried over anhydrous sodium sulfate and stored at 4 °C until further analysis.

The analytical samples were prepared by diluting an exactly weighed amount of essential oil (corresponding to 10 μL) with 1 mL of internal standard solution, previously prepared by diluting 0.7 mg of *n*-nonane to a total volume of 10 mL with cyclohexane. Such a preparation was repeated for each sample. These samples were directly used for the qualitative, quantitative, and enantioselective analyses.

The qualitative analysis was performed by injecting 1 μL of the EO solution (split 40:1) with the following thermal program. The initial oven temperature was 60 °C for 5 min, followed by a thermal gradient of 3 °C/min up to 180 °C and 15 °C/min up to 250 °C. The final temperature was maintained for 5 min. The injector temperature was set at 250°C, the carrier gas (helium) was set at 1 mL/min (constant flow). Additionally, a mixture of *n*-alkanes (C_9_–C_25_) was injected under the same conditions to determine the linear retention indices (LRIs).

The constituents of the essential oil were determined by comparison of mass spectra and LRIs, calculated according to van den Dool and Kratz [32], with tabulated data for DB-5ms [33] and literature for HP-INNOWax [20,21,22,23,24,25,26,27,28,29,30,31] (see Supplementary Material, Figures S3 and S4).

The quantitative analysis was performed by GC-FID, applying the same method and equipment conditions described above. Quantification was performed with external calibration curve and internal standard. Two calibration curves were obtained, injecting six dilutions of isopropyl caproate (calibration standard) and *n*-nonane (internal standard) in both columns. The dilutions were obtained by diluting 0.8 mg, 1.8 mg, 4.1 mg, 8.3 mg, 16.9 mg, and 34.5 mg of isopropyl caproate and an exactly weighed amount of 7.6–7.8 mg of *n*-nonane to 10 mL with cyclohexane. All calibration curves achieved a R^2^ > 0.995. The relative response factor (RRF) of each constituent of the EO versus isopropyl caproate was calculated, according to combustion enthalpy [36], prior to calibration.

The enantioselective analysis was performed by GC-MS on the same samples of the qualitative and quantitative analyses, according to the following method. The initial oven temperature was 60 °C for 2 min, followed by a thermal gradient of 2 °C/min until 220 °C, maintained for 2 min. Also, in this case, a mixture of *n*-alkanes (C_9_–C_25_) was injected under the same conditions to calculate LRIs. The elution order of the enantiomeric pairs was determined according to literature data for the same chiral selector [37].

## 5. Conclusions

The rare sesquiterpenoid Sipaucin A was isolated for the second time from a natural source. The spectroscopic evidence for the presence of some other terpenes of the Sipaucin family in this species has been obtained.

Furthermore, the essential oil of *S. echinata* was qualitatively and quantitatively characterized for the first time. A total of 27 and 19 compounds were identified and quantified in a non-polar and polar column, respectively. Major compounds were α-pinene (24.3%, 20.3%), β-pinene (21.7%, 22.7%), β-myrcene (11.3%, 14.8%), limonene (10.0%, 11.3%), *cis*-ocimene (8.5%, 8.1%), and *trans*-ocimene (8.9%, 8.4%). The enantioselective analysis performed on major monoterpenes indicated (+)-α-pinene as enantiomerically pure and (+)-β-pinene with 6.7% of enantiomeric excess.

## Figures and Tables

**Table 1:** Chemical composition of the essential oil distilled from *Siparuna echinata*.

Compounds	DB-5ms				HP-INNOWax				Reference Literature
	LRI ^a^	LRI ^b^	%	σ	LRI ^a^	LRI	%	σ	
α-pinene	932	932	24.3	2.36	1065	1076	20.3	2.69	[20]
camphene	947	946	0.9	0.18	1083	1092	0.8	0.43	[21]
sabinene	970	969	1.1	0.02	1120	1125	1.4	0.07	[21]
β-pinene	976	974	21.7	1.43	1108	1103	22.7	1.70	[22]
β-myrcene	989	988	11.3	2.34	1165	1161	14.8	2.57	[22]
limonene	1027	1024	10.0	3.55	1199	1194	11.3	3.94	[22]
*cis*-ocimene	1036	1032	8.5	1.77	1235	1228	8.1	1.40	[23]
*trans*-ocimene	1045	1044	8.9	5.43	1250	1244	8.4	5.10	[23]
perillene	1098	1102	0.1	0.03	-	-	-	-	-
linalool	1101	1095	0.6	0.60	1549	1546	0.8	1.04	[22]
nopinone	1138	1135	0.1	0.03	-	-	-	-	-
*trans*-pinocarveol	1140	1135	0.4	0.29	1643	1642	0.7	0.63	[24]
*cis*-verbenol	1146	1137	0.4	0.52	1668	1663	0.5	0.69	[25]
myrtenol	1196	1194	0.1	0.05	1789	1794	0.3	0.13	[26]
6-undecanol	1284	1284	0.2	0.10	1570	-	-	-	-
2-undecanone	1294	1284	0.2	0.13	1593	1598	0.4	0.60	[27]
decanoic acid	1376	1364	0.5	0.68	-	-	-	-	-
β-elemene	1387	1389	0.2	0.13	-	-	-	-	-
*trans*-caryophyllene	1415	1417	0.2	0.09	1580	1580	0.3	1.30	[28]
α-humulene	1451	1452	0.2	0.13	-	-	-	-	-
germacrene D	1477	1480	1.4	0.45	1687	1680	1.2	0.22	[23]
β-selinene	1484	1489	0.3	0.20	1703	1708	0.4	0.50	[29]
2-tridecanone	1496	1495	0.4	0.22	1808	1815	0.6	0.81	[30]
germacrene A	1502	1508	0.3	0.12	1748	1744	0.7	0.98	[23]
germacrene B	1554	1559	1.9	0.20	1814	1811	2.1	0.78	[31]
caryophyllene oxide	1578	1582	0.3	0.16	-	-	-	-	-
β-eudesmol	1651	1649	0.2	0.07	-	-	-	-	-
Monoterpene hydrocarbons			86.7				87.8		
Oxygenated monoterpenes			1.7				2.3		
Sesquiterpene hydrocarbons			4.5				4.7		
Oxygenated sesquiterpenes			0.5				0.0		
Others			1.3				1.0		
Total identified			94.7				95.8		

^a^ Calculated lineal retention index (LRI) according to van den Dool and Kratz [32]. ^b^ According to [33].

**Table 2:** Enantioselective analysis of *S. echinata* essential oil on diacethyl terbutylsilyl-β-cyclodextrin column.

LRIs	Enantiomers	Enantiomeric Distribution (%)	*ee* (%)
858	(+)-α-pinene	100.0	100.0
896	(+)-β-pinene	53.4	6.7
898	(-)-β-pinene	46.6	

**Table 3:** SIM/MS method for the detection of Sipaucins in the extract.

Sipaucin A		Sipaucin B		Sipaucin C	
Ion ^a^	Dwell Time (ms)	Ion ^a^	Dwell Time (ms)	Ion ^a^	Dwell Time (ms)
109	25	91	25	109	25
293	25	106	25	125	25
307	25	124	25	180	25
366 (M^+^)	25	142	25	293	25
		184	25	366 (M^+^)	25
		233	25		
		272	25		
		277	25		
		290	25		
		332	25		
		350 (M^+^)	25		

^a^ Electron impact ions according to [8].

## Supplementary Materials

The following are available online at https://www.mdpi.com/2223-7747/9/2/187/s1, Figure S1: ^1^H NMR (400 MHz) spectrum of Sipaucin A in CDCl_3_. Figure S2: ^13^C NMR (100 MHz) spectrum of Sipaucin A in CDCl_3_. Figure S3: Chemical composition of *Siparuna echinata* essential oil on a DB-5ms column. Figure S4: Chemical composition of *Siparuna echinata* essential oil on a HP- INNOWax column. Table S1: ^1^H NMR data (400 MHz, CDCl_3_) and ^13^C NMR data (100 MHz, CDCl_3_) of Sipaucin A (1).
