# Supplementary material for: Chemical Analysis of the Essential Oil from Siparuna echinata (Kunth) A. DC. (Siparunaceae) of Ecuador and Isolation of the Rare Terpenoid Sipaucin A
Source: Plants (Basel). 2020 Feb 4;9(2):187. doi: 10.3390/plants9020187 (PMC7076530; doi:10.3390/plants9020187)
Supplement: Supplementary file 1 [file plants-09-00187-s001.pdf]

Supplementary material

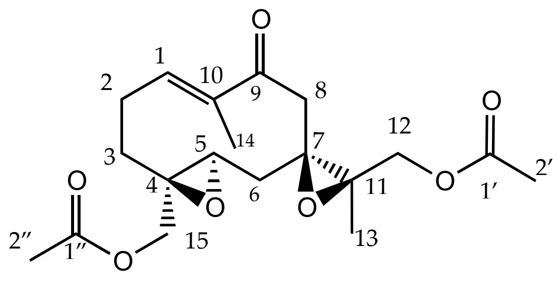

1

Sipaucin A (**1**). White powder; $C_{19}H_{26}O_7$; ^1H NMR (400 MHz, $CDCl_3$) δ 6.08 (1H, dq, *J* = 12.0, 1.6 Hz, H-1), 2.43 (m), 2.40 (m), 2.54 (m), 3.29 (1H, dd, *J*=9.2, 5.6 Hz, H-5), 2.73 (1H, dd, *J*= 15.2, 5.2 Hz, H-6), 1.37 (1H, dd, *J*=15.2, 6 Hz, H-6), 3.52 (1H,d, *J*=14.0 Hz, H-8), 2.89 (1H, d, *J*=14.0 Hz, H-8), 4.16 (1H, d, *J*=12.4, Hz, H-12), 4.12 (1H, d, *J*=12, Hz, H-12), 1.55 (3H, s, H-13), 1.88 (3H, d, *J*=1.6, Hz, H-14), 4.57 (1H, d, *J*=12.8, Hz, H-15), 3.68 (1H, dd, *J*=14.0, 1.6 Hz, H-15), 2.10 (3H, s, H-2′), 2.12 (3H, s, H-2″). ^{13}C NMR (100 MHz, $CDCl_3$) δ 138.4 (C-1), 34.6 (C-2), 32.3 (C-3), 61.8 (C-4), 61.5 (C-5), 24.2 (C-6), 60.8 (C-7), 42.9 (C-8), 202.0 (C-9), 136.7 (C-10), 62.2 (C-11), 65.1 (C-12), 16.0 (C-13), 13.0 (C-14), 63.2 (C-15).

Table S1. ^1H NMR data (400 MHz, $CDCl_3$) and ^{13}C NMR data (100 MHz, $CDCl_3$) of Sipaucin A (1)

Position	1	C	1
**1**	6.08 (1H, dq, *J* = 12.0, 1.6 Hz)	**1**	138.4
**2a**	2.43m	**2**	34.6
**2b**	2.40 m	**3**	32.3
**3**	2.54 m	**4**	61.8
**5**	3.29 (1H, dd, *J*=9.2, 5.6 Hz)	**5**	61.5
**6a**	2.73 (1H, dd, *J*= 15.2, 5.2 Hz)	**6**	24.2
**6b**	1.37 (1H, dd, *J*=15.2, 6 Hz)	**7**	60.8
**8a**	3.52 (1H, d, *J*=14.0 Hz, H-8)	**8**	42.9
**8b**	2.89 (1H, d, *J*=14.0 Hz, H-8)	**9**	202.0
**12a**	4.16 (1H, d, *J*=12.4, Hz)	**10**	136.7
**12b**	4.12 (1H, d, *J*=12, Hz)	**11**	62.2
**13**	1.55 (3H, s)	**12**	65.1
**14**	1.88 (3H, d, *J*=1.6, Hz)	**13**	16.0
**15a**	4.57 (1H, d, *J*=12.8, Hz)	**14**	13.0
**15b**	3.68 (1H, dd, *J*=14.0, 1.6 Hz)	**15**	63.2
**2′**	2.10 (3H, s)	**-**	-
**2″**	2.12 (3H, s)	**-**	-

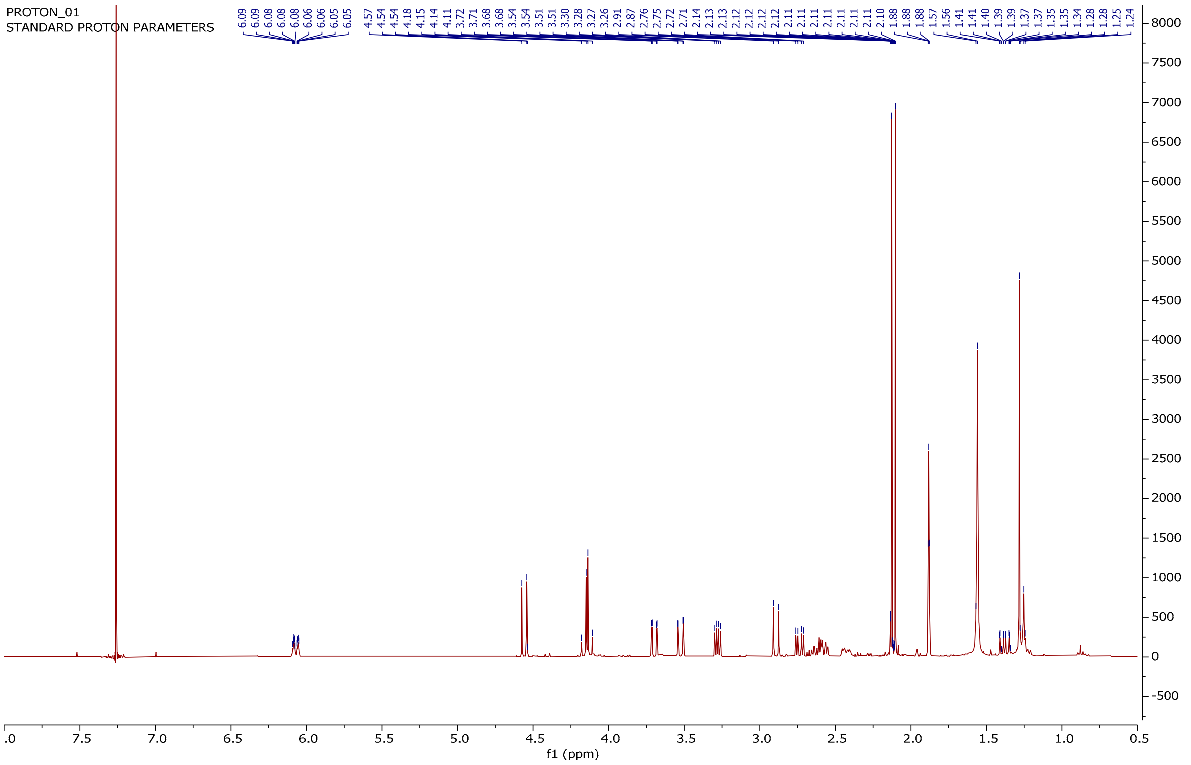

Figure S1. ^{1}H NMR (400 MHz) spectrum of Sipaucin A (**1**) in $CDCl_3$

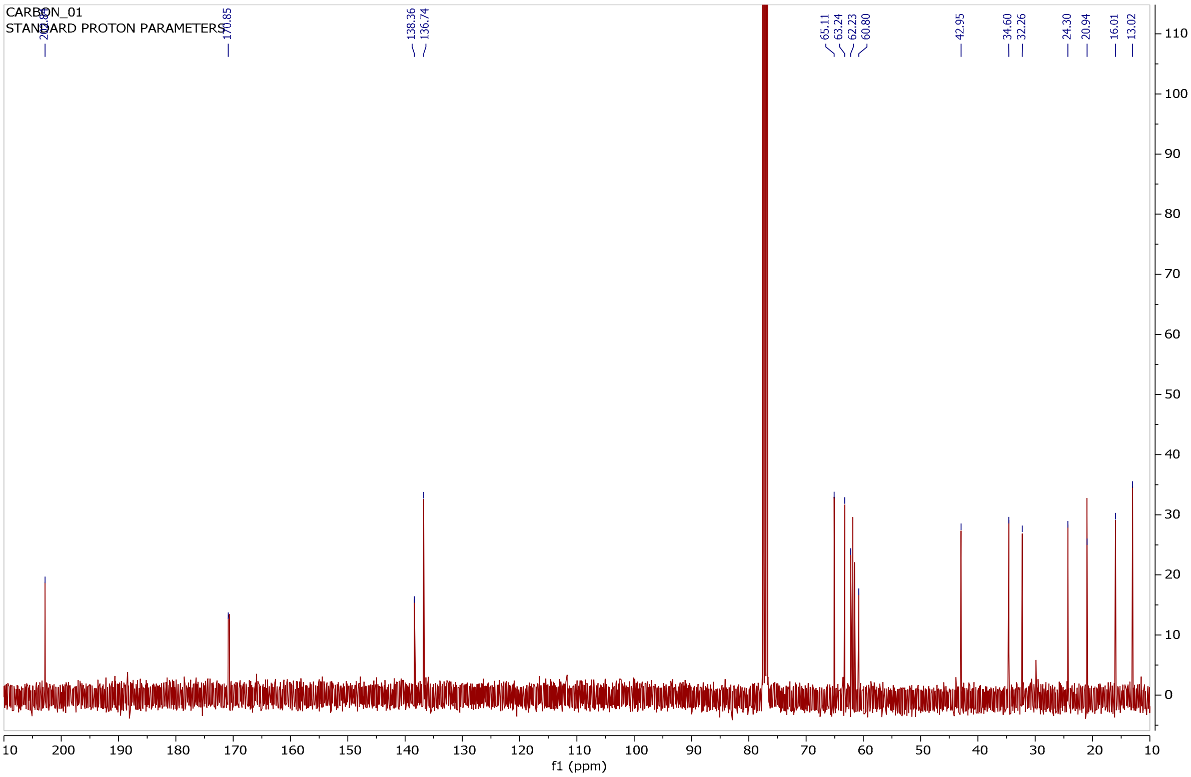

Figure S2. ^{13}C NMR (100 MHz) spectrum of Sipaucin A (**1**) in $CDCl_3$.

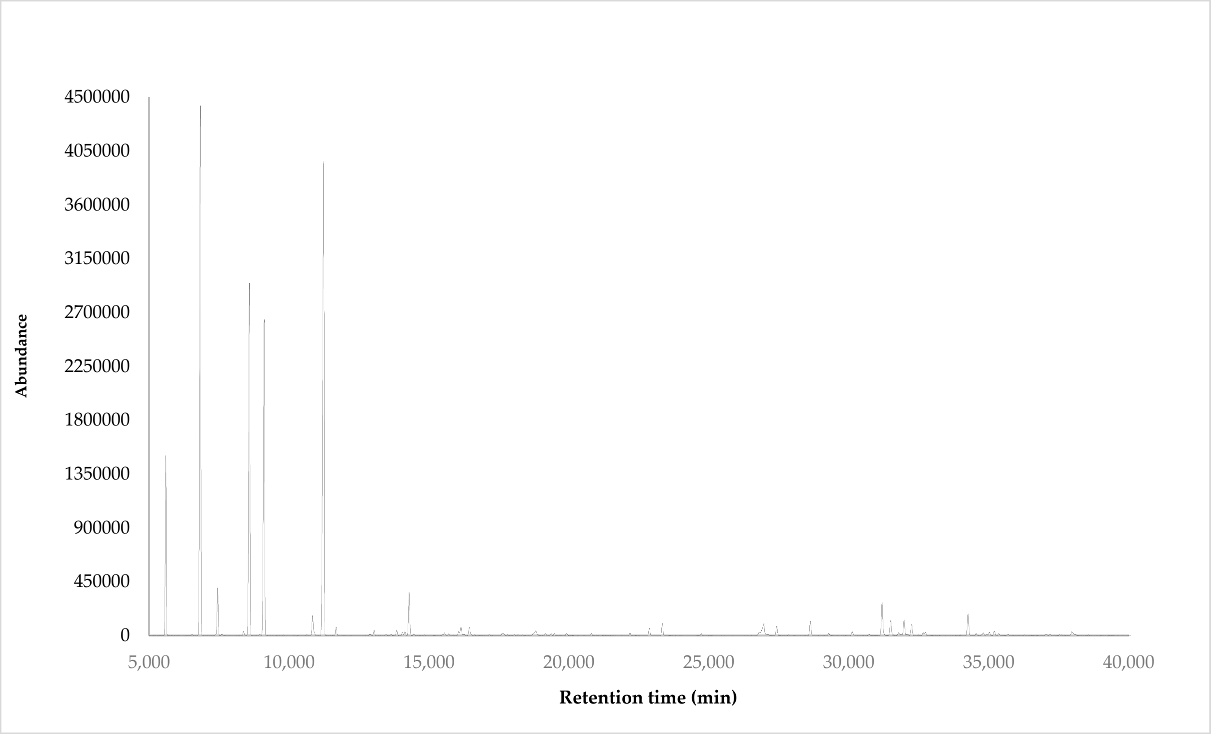

Figure S3. Chemical composition of *Siparuna echinata* essential oil on a DB-5ms column.

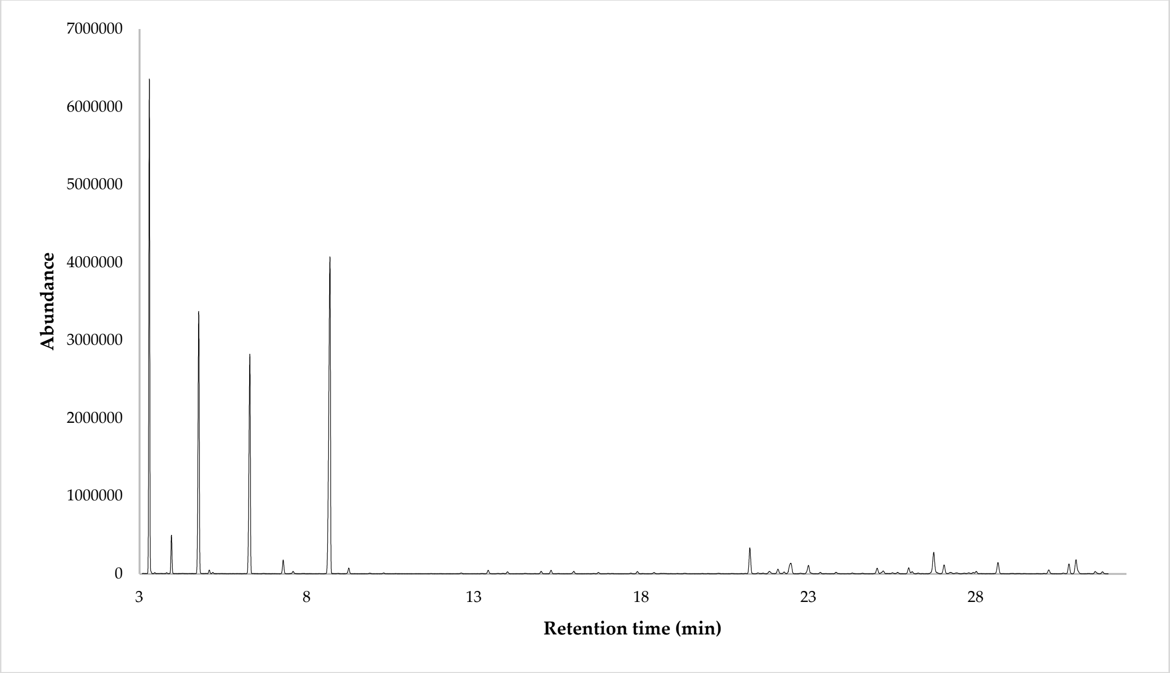

Figure S4. Chemical composition of *Siparuna echinata* essential oil on an INNOWax column.
